# Deregulation of apoptotic proteins by induction of *Dendropthae falcata* (L.f.) Ettingsh plant extract in breast cancer cells: A proteome-wide analysis

**DOI:** 10.22038/IJBMS.2023.71125.15450

**Published:** 2023

**Authors:** Alwin Beschi Durairaj, Ananthi Sivagnanam, Reginald Appavoo Monikam, Rajapandiyan Krishnamoorthy, Mohammad Z. Ahmed, Ali S. Alqahtani, Ponnani Kaja Mydeen

**Affiliations:** 1 Department of Botany & Research Center, Scott Christian College, Nagercoil – 629003, Affiliated to Manonmaniam Sundaranar University, Abishekapatti, Tirunelveli – 627012, Tamil Nadu, India; 2 Department of Proteomics, Clinbiocare Technology, Tenkasi – 627 814, Tamil Nadu, India; 3 Centre of Molecular Medicine and Diagnostics (COMManD), Saveetha Dental College and Hospitals, Saveetha Institute of Medical and Technical Sciences, Saveetha University, Chennai, India; 4 Department of Food Science and Nutrition, College of Food and Agriculture Sciences, King Saud University, Riyadh 11451, Kingdom of Saudi Arabia; 5 Department of Pharmacognosy, College of Pharmacy, King Saud University, Riyadh, Saudi Arabia; 6 Department of Pharmacology and Toxicology, University of Mississippi Medical Center, Jackson, Mississippi, USA

**Keywords:** 2D-gel electrophoresis, Breast cancer cell line, Dendropthae falcate, Mass spectrometry, Medicinal plant, Proteomics

## Abstract

**Objective(s):**

The present study evaluated the protein-based analysis to unravel the role and mechanism behind the *Dendropthae falcata* plant extract treatment in breast cancer cells.

**Materials and Methods:**

The protein sample was extracted from the cancer cells after treatment with the plant extract and subjected to two-dimensional electrophoresis for protein separation. Further, the proteins that were differentially regulated among the samples which were treated and non-treated were selected and processed further for protein identification using a tandem mass spectrometry approach.

**Results:**

Using these strategies, we identified 16 potential candidates which were showing remarkable changes in treated samples. All the candidates were analyzed further for gene ontology analysis, and it was observed that all proteins were involved in multiple pathways pertaining to the carcinogenesis process. Specifically, apoptotic pathway proteins including BAD, BIK, BID, CASP8, MCL1, BCL2, and BAK1 were highly impacted by treatment with *D. falcata *plant extract. All these protein hits were further taken for validation experiments using RT PCR analysis.

**Conclusion:**

Initiation of these apoptotic proteins by *D. falcata* plant extract treatment in breast cancer cells shows a positive direction toward nature-based alternative medicine.

## Introduction

Breast cancer is known to be a major problem for most women. It is observed that in the current scenario, the number of new cases is getting increased. It is reported that in 2018, the mortality rate was very high including 5 lakh deaths (1). Breast cancer is known to be the most common cancer in India followed by other cancers. In recent years, more numbers of new cases were reported, and the total percentage contributes to about a quarter part of total cancers, especially in Indian women. It is also observed that approximately more than ten percent of cancer-related mortalities were recorded because of breast cancer (2). As per the age criteria, in Indian women, it is observed that most of the women were diagnosed in the age range of 40–50 years in cities. With respect to rural areas, the age range differs from 65–70 (3, 4). A recent study found that in the northern part of India, about 26% of women were diagnosed with breast cancer under the age of 35 (5). Overall, breast cancer in India is very well known for high mortality with poor survival success. All advanced treatment procedures were known to induce various side effects which give more trouble to the patient. As well, till now there is a huge drawback in the treatment protocols as it is not leading to improved survival of the patient. All chemo and radiology-based treatments lead to severe problems such as losing strength and immunity. It also led to heavy weight loss and hair loss. To overcome these problems, an efficient method is required to treat this type of cancer condition. This will eventually help the patients to get cured without major side effects.

To find a better solution for all these problems, recently, researchers have started focusing on alternative treatment protocols using plant-based compounds. Studies proved that all these plant-based compounds have beneficial effects in enhancing the survival of patients without inducing any major side effects.

*Dendropthae falcata* plant is traditionally applied to treat various types of health problems such as ulcers, asthma, impotence, paralysis, skin diseases, and wounds. Most of the studies proved that this plant extract exhibits potent anti-oxidant activity by inhibiting lipid peroxidation, reducing glutathione and superoxide dismutase levels, and increasing catalase activity. 

*D. falcata *belongs to the *Plantae* kingdom with the *Tracheophyta phylum *and* Magnoliopsida* class. It belongs to the Santalales order and the *Loranthaceae* family. *D. falcata *is well known for its traditional medicinal properties, especially in India and Indonesia. They were used to treat wounds, ulcers, asthma, paralysis, fertility, skin diseases, menstrual disorders, and so on (6). Hence, these plants were available in the Ayurvedic system of medicine and other ethnomedicinal systems. Mostly, these plant products were consumed with other ingredients to make edible tea, decoction, or plaster to treat human diseases, while raw materials are fed to livestock to improve growth performance (7).

Hence, we focused on analyzing the effects of *D. falcata *in breast cancer cell lines. In this paper, we aimed to understand the molecular level response of these plant compounds in treating breast cancer cell lines. These types of studies will help us to unravel the novel proteins which are getting altered during the treatment with plant compounds. 

Proteomic analysis and protein-based studies are getting their significance in current scenarios which help us to understand the network of bioprocess involved in the carcinogenesis process. In this study, we aim to evaluate the significance of *D. falcata *in treating breast cancer cells using proteome-wide analysis.

## Materials and Methods

***Collection of plant samples***

The whole plant of *D. falcata* was collected around the Kavalkinaru area at the latitude 8.2765° N and longitude 77.5808° E, Tirunelveli District, Tamil Nadu. The collection was made between June 2018 and August 2018. Polythene bags were used to collect plant materials in fresh condition. All the collected plants were trimmed off with scissors. Leaves were separated from the plant twigs. Further, they were kept for drying at room temperature. After drying, leaves were crushed with the help of an electrical stainless-steel blender and sieved using thin nylon mesh to get a fine powder. 

The collected plant’s morphology was used for identification purposes. The plant was identified and authenticated by the Botanical Survey of India, Coimbatore with the plant authentication certificate no BSI/SRO/5/23/2019/Tech/462. The plants were cleaned well with running water and this procedure helped to eliminate the epiphytes and necrotic parts. In addition, in order to eliminate the debris, the plants were cleaned twice with tap water. After cleaning the collected plants were shade dried for a week at 30 °C. 

Further, the plant samples were extracted with 70% methanol using Soxhlet extraction for 24 hr. Evaporation was through reduced pressure and resuspending the obtained mass in double distilled water with a concentration of 100 mg/ml.

***Cell culture***

The MCF-7 cell lines were obtained from the National Centre for Cell Sciences (NCCS), Pune, India. For cell maintenance, Dulbecco`s Modified Eagle Media was adapted with ten percent fetal bovine serum. Antibiotics such as Penicillin (100 U/ml), and streptomycin (100 μg/ml) were used along with the media. The cell lines were kept in appropriate humid conditions with 5% CO_2_ at 37 °C.

***MTT assay***

The cytotoxicity assay of the selected plant materials on MCF-7 cells was performed as described earlier (8, 9). The reduction of the MTT reagent was done by the viable cells through their mitochondrial dehydrogenase enzyme. Eventually, this process results in purple color formation which will be analyzed further by dissolving in appropriate solutions and absorbance is measured at 540 nm by a plate reader. The MTT assay was performed for the untreated cancer cells and *D. falcata* treated cancer cells as described above with the concentration range from 50 µg to 350 µg. Further, IC_ 50_ value was calculated based on the MTT analysis.

***Sample preparation method for proteomic analysis***

The protein analysis was performed for the untreated cancer cells and *D. falcata *treated cancer cells as described below. For lysis buffer protein extraction, the cell lines after appropriate treatment with plant extracts were treated with 7M urea, 2M thiourea, 4% w/v 3- [(3-holamidopropy/dimethy-ammonia]-1- propane sulfonate (CHAPS) (Pierce, Rockford, IL, USA), 0.2% ampholite 3–10 (Amersham, USA), 100 mM PMSF, and 100 mM DTT. The mixture was sonicated. Following the process, the sample was centrifuged at 15,000 rpm for 1 hr at 4 °C and the supernatant was reserved, aliquoted, and stored. 

***Two-dimensional gel electrophoresis***

*Passive rehydration*

Samples in rehydration buffer were applied to the slots in the dry strip re-swelling tray, followed by placing of the IPG strips (GE Healthcare) and addition of 3.5 ml of IPG cover strip fluid on top of the strip. Rehydration was done for a minimum of 12 hr.

*Isoelectric focusing and equilibration*

Rehydrated strips were kept for focusing at 20 °C as per standard protocols prescribed by manufacturer instructions. The focusing step was carried out for 13 hr, and further, the strips were frozen at -80 °C overnight. The next day, the stored strips were thawed to ambient temperature and strips were equilibrated with IAA and DTT in 2 different steps.

*Second dimension SDS-PAGE*

After completing equilibration, the strips were taken and subjected to SDS-PAGE analysis. After inserting the plates in the DALT six apparatus (GE Healthcare), used for second-dimension electrophoresis, the bottom of the upper chamber was sealed with 1% agarose in 2X electrophoresis buffer. The upper chamber was then filled with 2X electrophoresis buffer. The second dimension run settings for six gels were 600V, 400mA, and 12W for 45 min and then 600V, 400mA, and 60W for 5 hr.

*Colloidal coomassie staining*

The protocol of Candiano and his group (2004) (3) was followed. Fixation of gels was performed with methanol and acetic acid in a ratio of 4:1 in H_2_O and kept for 60 min. Then, the gels were rinsed with H_2_O thrice with 10 min of incubation for every change of H_2_O. Finally, gels were placed in Coomassie brilliant blue staining solution and kept for overnight incubation. Post-staining, the gels were placed in H_2_O for the destaining process.

***Processing of 2DE spots for MALDI TOF mass spectrometry***

Selected protein spots from a stained 2D gel were excised manually using a 2.5 mm diameter metal punch. Using a sterile blade, the gel spot was cut into 1 mm pieces. The gel pieces were rinsed thrice in H_2_O followed by a destaining process using 25 mM NH_2_HCO_3_ which was prepared in ACN. The process was repeated until the color disappeared completely. Further, the destained gel pieces were subjected to a dehydration process with ACN and the process was incubated for 20 min. It was followed by the drying step using a vacuum for half an hour. After the dehydration process, the gel pieces were subjected to a rehydration step with 0.4 mg of trypsin (Promega), prepared in 100 mM NH_2_HCO_3_ in ACN. Furthermore, the gel pieces were treated with 40 mM NH_2_HCO_3 _that was prepared in ACN and incubated for 16 hr. 

After incubation, the tubes were spun shortly and the sup solution was saved. Further, the gel pieces were processed with 20 μl of 0.1% trifluoroacetic acid (TFA) made in sixty percent of ACN to extract the peptides. After 10 min of incubation, the tubes were spun shortly and the sup solution was saved and added to the earlier stored sup solution. Finally, the gel pieces were treated with 20 μl 100 % ACN and kept for 10 min of incubation. After incubation, the tubes were spun to obtain the sup solution which was added to the earlier stored supernatants. All the stored supernatants were subjected to vacuum drying for one hour. The extracted dried peptides were directly suspended in the 0.1 % formic acid and further taken for mass spec analysis.

***Mass spectrometry analysis***

The extracted peptides were analyzed through mass spectrometry using SHIMADZU MALDI 7090 (TOF/TOF MS). The acquisition parameters were as follows: the lyophilized samples were diluted in 8 μl of 50:50 (ACN: Water in 0.1 FA). Then samples were vortexed and spotted about 0.7 μl in volume to the stainless-steel target plate, and the CHCA matrix prepared in 50/50 (ACN/Water in 0.1% TFA) concentration was spotted above the sample spot and air dried. The analysis was carried out in Reflection Mode with the mass range of 1–5000 Da, eliminating the Matrix ions through setting up the ion-gate blanking mass of 700 Da (ions below the 700 mz were not acquired by the system). The laser energy was set to 35–45 (based on the sample) with a laser beam diameter of 100 microns and pulsed extraction at 1500 mz mass (focusing ions at a range). The processing parameters were as follows: the subtract baseline using the filter width feature allows us to move the baseline and improves the signal-to-noise ratio and resolution of the peaks. The value set for processing was 12. Smoothing reduces the “spikiness” of data caused by background electronic noise. Data were processed with the Gaussian smoothing method: The value set for processing was 3. Peak delimiting method for determining the peak Mass: threshold centroid of 25% is used for the processing. Mono isotopic mass picking was from the 700–3000 range with a minimum of two isotopes.

***Gene ontology by PANTHER software and protein interaction by STRING***

Analysis of functional enrichment of differentially regulated proteins was performed in the PANTHER software (10) and protein interaction studies were performed using the STRING database (11). 

***Quantitative real-time PCR (qPCR)***

RNA was isolated from untreated and treated MCF-7 cell lines using an RNeasy kit (Qiagen, USA) and the protocol was followed as provided by the manufacturer. The qPCR assay was performed with SYBR-Green mix (Qiagen, USA) on Rotor-Gene Q 5plex HRM Platform (Qiagen, USA). The mRNA expression of the seven target genes was analyzed and the details were provided in Table 1. The profile pattern was adapted from standard protocol which includes 95 °C – 180 seconds; cycling (40) with 95 °C – 30 sec; 58.1 °C – 20 sec; 60 °C – 20 sec. The difference in the fold change was calculated based on the comparative threshold cycle (Ct) method. For relative quantification, beta-actin was kept as a control gene to normalize the expression levels. All experiments were performed with three sets of samples for each target and the 2-ddct method was applied to identify the expression level.

## Results

***MTT assay***

**Figure 1:**
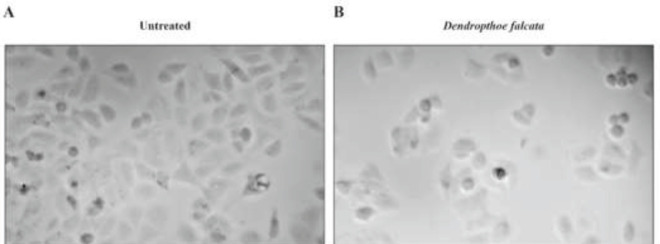
Cell viability assay of untreated and Dendropthoe falcata plant extract treated breast cancer cells

**Figure 2:**
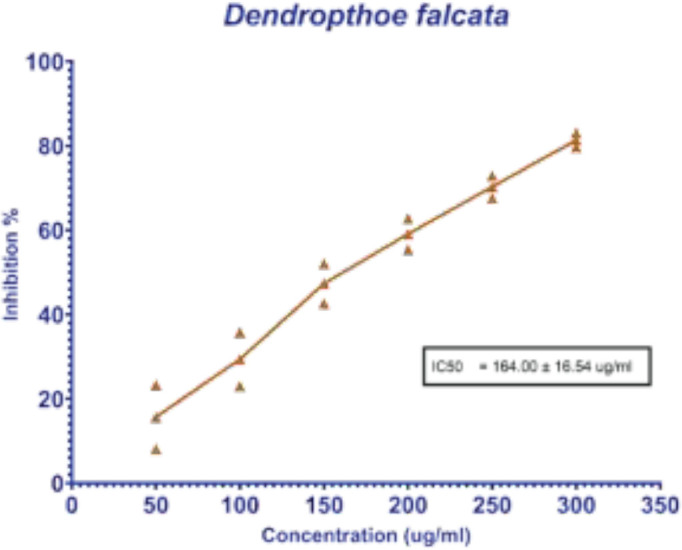
IC_50_ values of *Dendropthoe falcata* plant extract treated cells

To evaluate the effect of *D. falcata *plant extract against the breast cancer cell line, the cell viability was analyzed by MTT assay. The data suggest that the dose of 164 ug/ml was effective in eradicating breast cancer cells. This clearly describes that the plant extract has effective biological components that have efficient activity in killing cancer cells. It was observed that the morphological structures were prominently changed in the treated cancer cells. The changes were represented in Figure 1 and the data about dosage were represented in Figure 2.

***2D gel electrophoresis and protein identification by mass spectrometry***

**Figure 3:**
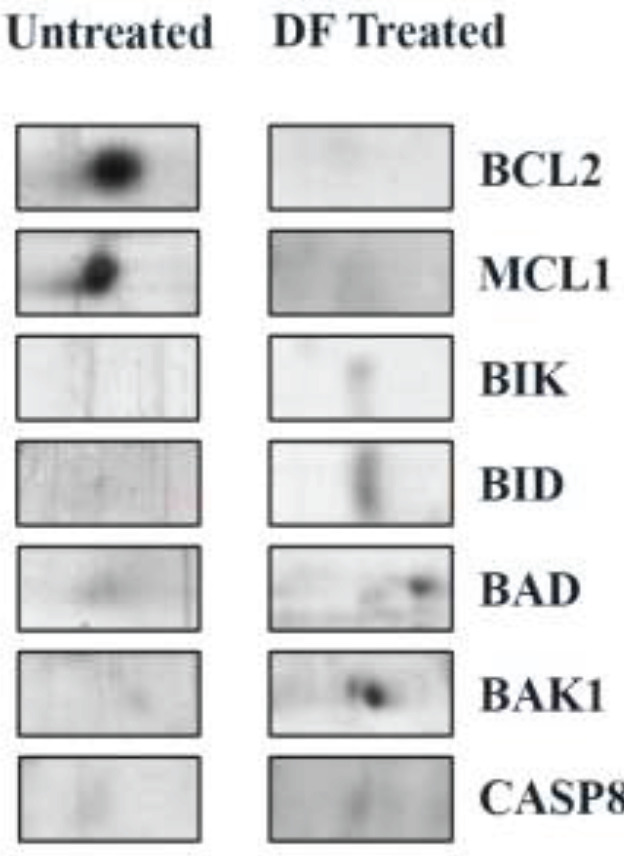
Representation of magnified 2D gel image focusing on all seven differentially expressed proteins in both untreated control cells and *Dendropthoe falcata *(DF) treated cells

To understand the protein expression alterations, the treated and untreated cancer cells were subjected to protein extraction procedures. Further, the protein samples were taken for 2D electrophoresis analysis, and 2D images focusing on the differential expression protein spots were represented in Figure 3. Both 2D images were compared and analyzed in Image analysis software (Table 2) and sixteen differentially regulated protein spots were further subjected to mass spectrometry analysis for protein identification. Among 16, eight were up-regulated and eight were down-regulated in the breast cancer cells upon treatment with the plant extract. All data about protein spots, their spot intensity, and the analysis were represented in Table 2.

To determine the protein profiling of breast cancer cells MCF7 upon treatment with *D. falcata *extract, 2D electrophoresis was performed (Figure 2). Sixteen differentially regulated protein spots were analyzed by image master analysis and represented in the class analysis table in Table 2. Among 16, eight were up-regulated and eight were down-regulated in the breast cancer cells upon treatment with the plant extract. Table 2 describes the spot intensity values of the spots from the control and treated group with fold change and ANOVA value. Most of the differentially regulated protein spots were found to be statistically significant. All the protein spots were identified by tandem mass spectrometry and the details were represented in Table 3 with other relevant details. 

***Bio informatic analysis of the identified proteins***

**Figure 4:**
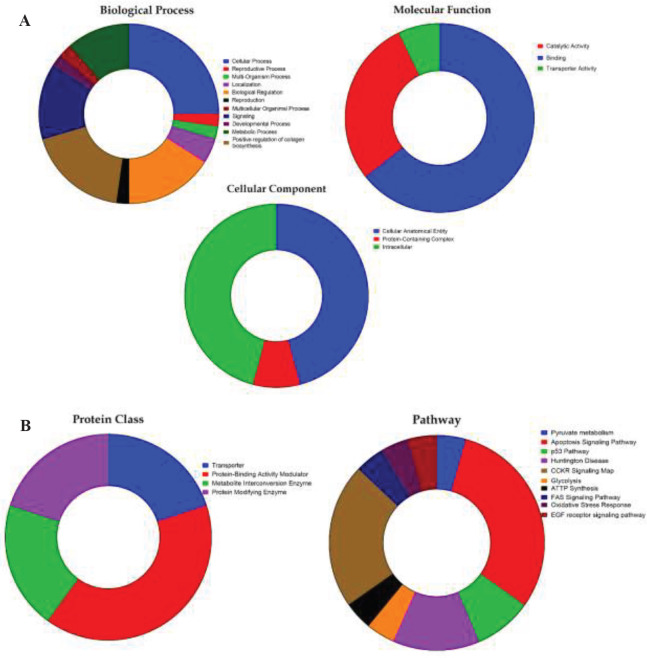
Gene ontology analysis for the identified set of proteins analyzed in the PANTHER database

Gene ontology was performed for all identified proteins using the PANTHER database and represented in Figures 4A and 4B. Protein was categorized based on its functional properties such as molecular function, cellular component, and biological process and represented in Figure 4A; protein class and pathway analysis were represented in Figure 4B. With respect to molecular function, most of the identified differentially regulated proteins were observed to have binding activity, followed by catalytic activity and transporter activity. In cellular component analysis, an equal percentage of proteins showed cellular anatomical entity and intracellular region followed by a protein-containing complex. The biological process analysis revealed that most of the differentially expressed proteins have a cellular process, followed by biological regulation, positive regulation of collagen biosynthesis, signaling, metabolic process, and localization. All the identified proteins were categorized into four protein classes including protein modifying enzyme, protein binding activity modulator, transporter, and metabolite interconversion enzyme. With respect to pathway analysis, ten pathways were observed and most of them were involved in the tumorigenesis process hence it indicates that all the differentially expressed proteins play a major role in cancer metabolic pathways such as apoptosis signaling pathway, CCKR signaling pathway, p53 pathway, FAS and EGF signaling pathway. 

***Protein interaction analysis of the identified proteins***

**Figure 5:**
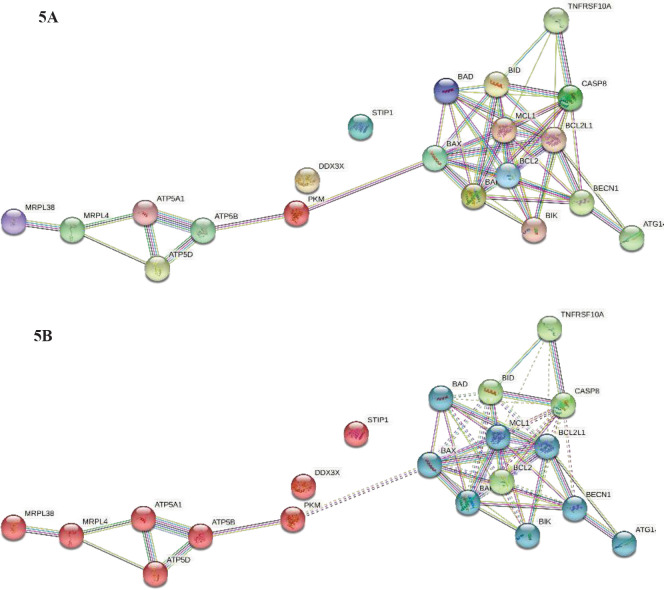
Protein interaction analysis for the identified proteins using the STRING database

STRING analysis was performed for all the identified proteins and represented in Figure 5A. Among the identified protein’s interaction, two proteins including DDX3X and STIP1 were not found to be interacting with other proteins. They are observed to be individual players in the treatment responses with the plant products in the breast cancer cell line. Other identified proteins such as BIX, BAD, BID, MCL1, BCL2, BIK, ATG1, BECN1, BCL2L1, CASP8, TNFRSF10A, and BAK1 were shown to have strong interaction among themselves, as well as interaction with other groups of proteins such as ATP family proteins and MRP family proteins. Interestingly PKM was shown to interact with both networks. On the whole, this protein interaction analysis depicts the significance of the identified proteins which were differentially regulated upon treatment with the plant extracts in the MCF7 cell line. With respect to STRING cluster analysis, three major groups were formed, and cluster analysis was represented in Figure 5B.

***mRNA expression analysis for apoptotic genes***

**Figure 6.**
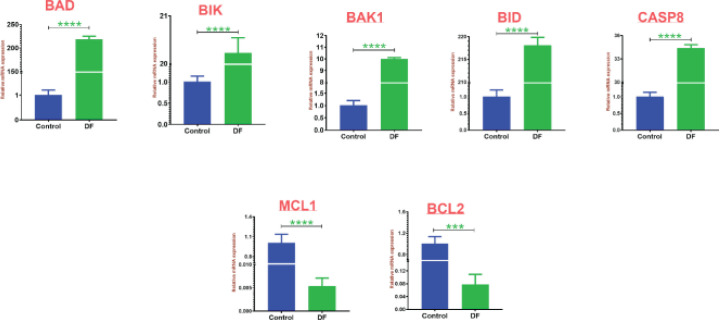
RT PCR analysis for BAD, BIK, BAK1, BID, CASP8, MCL1, and BCL2 upon plant extract treatment against breast cancer cells

Among the identified proteins, BAD, BIK, BAK1, BID, CASP8, MCL1 and BCL2 proteins were further validated using quantitative real-time PCR. The breast cancer cell line, MCF7 was treated with *D. falcata *plant extract and incubated for 24 hr, furthermore, RNA was extracted and subjected to cDNA synthesis, and qRT PCR was performed. The data was represented in Figure 6. With coherence to the proteomics results, mRNA expression was also shown to have down-regulation of two targets (MCL1 and BCL2) and up-regulation of five targets, including BAD, BIK, BAK1, BID, and CASP8. All the differentially regulated proteins were statistically significant.

**Table 1:** RT PCR Primer Sequences for selected gene targets

**Gene**	**Primer**	**Sequence（5’ to 3’）**
BAD	Forward	GAGTGAGCAGGAAGACTCCAGC
	Reverse	TCCACAAACTCGTCACTCATCC
BIK	Forward	GTCATGCCAAGAACCTCCAT
	Reverse	GGTGGCTTACAGACGCTGC
BAK1	Forward	TTTTCAGGTCTCAGTGGAGGA
	Reverse	CATTCCTGGAAACTGGGCT
BID	Forward	CAGCTCCGACTCACTCCTG
	Reverse	ACAAATACGAATGTGCAGCG
CASP 8	Forward	AGAGTCTGTGCCCAAATCAAC
	Reverse	GCTGCTTCTCTCTTTGCTGAA
MCL1	Forward	GATGATCCATGTTTTCAGCGAC
	Reverse	CTCCACAAACCCATCCCAG
BCL2	Forward	AGGAAGTGAACATTTCGGTGAC
	Reverse	GCTCAGTTCCAGGACCAGGC
ACTIN	Forward	GATGATGATATCGCCGCGCT
	Reverse	CCTCGTCGCCCACATAGGAA

A) Untreated breast cancer cells. B) *Dendropthoe falcata *plant extract treated breast cancer cells

**Table 2:** Class analysis table representing the details of the differentially regulated protein spots

**Gene ID**	**Protein name**	**Control**	**DF**	**Anova**	**Regulation status upon treatment**
BCL2	Apoptosis regulator Bcl-2	5.89856	-	1.80E-07	Down
BCL2L1	Bcl-2-like protein 1	7.82454	-	7.40E-04	Down
BAX	Apoptosis regulator BAX	1.41626	2.73783	2.49E-04	Up
MCL1	Induced myeloid leukemia cell differentiation protein Mcl-1	8.3055	-	0.0210668	Down
BCL2L1	Bcl-2-like protein 1	2.48005	-	4.25E-08	Down
BIK	Bcl-2-interacting killer	0.33967	1.34187	0.0119247	Up
BID	BH3-interacting domain death agonist	0.422722	3.06447	5.30E-05	Up
BAD	Bcl2-associated agonist of cell death	1.35919	5.88453	0.0316357	Up
BAK1	Bcl-2 homologous antagonist/killer	1.03755	4.57527	0.0381953	Up
BECN1	Beclin-1	0.665779	6.21158	3.68E-05	Up
CASP8	Caspase-8	0.170338	2.43937	5.73E-05	Up
MRPL38	39S ribosomal protein L38, mitochondrial	1.18098	1.74821	0.25067	Up
DDX3X	ATP-dependent RNA helicase	1.37135	-	9.13E-05	Down
STIP1	Stress-induced-phosphoprotein 1	0.435198	-	0.312141	Down
ATP5F1A	ATP synthase subunit alpha, mitochondrial	13.5705	-	2.62E-12	Down
PKM	Pyruvate kinase PKM	2.57312	-	0.0188901	Down

DF: *Dendropthoe falcata*

**Table 3:** Protein identification table depicting the data about the identified proteins

**Match ID**	**Gene ID**	**Accession No**	**Protein name**	**Mol weight**	**pI**
0	BCL2	P10415	Apoptosis regulator Bcl-2	26.265	6.75
1	BCL2L1	Q07817	Bcl-2-like protein 1	26.048	4.86
2	BAX	Q07812	Apoptosis regulator BAX	21.184	5.08
3	MCL1	Q07820	Induced myeloid leukemia cell differentiation protein Mcl-1	37.337	5.51
4	BCL2L1	Q07817	Bcl-2-like protein 1	26.048	4.86
5	BIK	Q13323	Bcl-2-interacting killer	18.015	4.21
6	BID	P55957	BH3-interacting domain death agonist	21.995	5.25
7	BAD	Q92934	Bcl2-associated agonist of cell death	18.392	6.6
8	BAK1	Q16611	Bcl-2 homologous antagonist/killer	23.409	5.66
9	BECN1	Q14457	Beclin-1	51.896	4.83
10	CASP8	Q14790	Caspase-8	55.391	4.99
11	MRPL38	Q96DV4	39S ribosomal protein L38, mitochondrial	44.596	7.19
12	DDX3X	O00571	ATP-dependent RNA helicase	73.243	6.73
13	STIP1	P31948	Stress-induced-phosphoprotein 1	62.639	6.4
14	ATP5F1A	P25705	ATP synthase subunit alpha, mitochondrial	59.75	9.16
15	PKM	P14618	Pyruvate kinase PKM	57.936	7.96

A) Functionally the proteins were categorized based on their biological process, molecular function, and cellular component. B) Proteins are classified based on their properties and pathways

5A represents the whole analysis, and 5B represents the cluster analysis

## Discussion

***Bcl-2***

B-cell lymphoma-2 (Bcl-2) is the primary candidate included in the Bcl-2 group of apoptotic regulatory proteins that were involved in assisting oncogenesis by enabling apoptotic resistance. To date, over 15 proteins have been identified to show homology with the Bcl-2 homology domain and are classified into pro-survival, pro-apoptotic, and BH3-only proteins (12). 

Bcl-2 is known to be expressed in normal and breast cancer cells and is reported to be overexpressed when the cells are treated with estrogen. The expression of Bcl-2 in breast cancer is also favorably linked with differentiated and prognostic markers, including ER/PR expression, slow proliferation, small tumor size, and HER2 negativity. A few studies have considered the use of Bcl-2 as a prognostic marker for breast cancer. Bcl-2 overexpression was observed exclusively in ER+ disease, but the pathological function is not clear (13). Teixeira and coworkers showed that the MCF-7 breast cancer cells had enhanced sensitivity to the cytotoxic drug doxorubicin when treated with anti-sense Bcl-2 (14). Furthermore, a group of BH3-mimicking small molecules that functions like the pro-apoptotic BH3-only protein was designed and utilized to counteract Bcl-2, Bcl-XL, and Bcl-W anti-apoptotic proteins’ effects (15). 

In our study, Bcl-2 was expressed in control cells. When treated with the DF compounds, the Bcl-2 spot was not found on the 2D-electrophoresis analysis. DF treatment showed that the expression of Bcl-2 was inhibited. This massive down-regulation of the anti-apoptotic Bcl-2 by the DF test compounds, along with improved apoptotic rates in these samples, could explain the therapeutic value of these compounds for cancer therapy.

***Bcl2-L1***

BCL2-L1 (Bcl-2 like-1) is a member of the BCL-xL family as well as a member of the Bcl-2 family of anti-apoptotic proteins. It has the ability to trigger the process of apoptosis by having affinity to and further to inhibit VDAC and by this step it arrests the release of mitochondria for the caspase activator CYC1. Alternate splicing could lead to Bcl-xL (anti-apoptotic) or Bcl-xS (pro-apoptotic) (16). Bcl2-L1 shares most of its functions and features with Bcl-xL and is known to be seen with abundant expression in a wide range of cancers. (17). Glioblastoma cell lines such as U251 and u87 when treated with miR-342 resulted in the reduction in the expression of anti-apoptotic genes Bcl2-L1 and Mcl-1. Overexpression of miR-342 resulted in enhanced apoptosis and decreased Bcl2-L1 proteins in glioblastoma cells (18). Expression of Bcl2-L1 was shown to be correlated to 20 q gain in colorectal cancer cell lines. The protein expression was enhanced, while the mRNA did not show any change in the expression of Bcl2-L1. Thus, the expression could be regulated at the post-transcriptional level by specific factors on the 20 q amplicon (19). Specifically, it was observed that Bcl2-based drugs were observed to exhibit reduction in gastric cancer tumor size (20). These findings prove that Bcl2 possesses unique parameters in treating various cancers.

The role of Bcl2-L1 in breast cancer is yet to be investigated. In our study, untreated breast cancer cell lines were shown to express high amounts of Bcl2-L1, whereas cell lines treated with the test compounds showed no expression of Bcl2-L1. This means that DF compounds tested in our study showed an outstanding potential to inhibit anti-apoptotic proteins’ expression, promoting cell death in these cancer cell lines.

***Mcl-1***

Induced myeloid leukemia cell differentiation protein (Mcl-1) is also a member of the Bcl-2 family of anti-apoptotic proteins that help resist apoptosis by interacting with pro-apoptotic proteins inhibiting the release of pro-apoptotic factors such as cytochrome C (21). The expression of anti-apoptotic proteins such as Bcl-2 and Mcl-1 is tightly regulated at several levels starting from transcriptional regulation to post-translational modifications. Mcl-1 expression could be regulated by JAK/STAT, MEK/ERK, p38/MAPK, and PI3K/AKT pathways (22, 23), and micro RNAs such as miR-29 b and miR-125 b (24). Mcl-1 was found to have a meager half-life of around 30 min in a cell, and the stability of Mcl-1 is strictly regulated by polyubiquitin ligases such as Mcl-1 ubiquitin ligase E3, which helps in protein degradation (25), and deubiquitinases such as USP9X and USP13, which helps in enhancing the stability of Mcl-1 (26). This tight regulation of Mcl-1 expression is altered significantly in several cancers.

Mcl-1 was observed to be getting increased in a different range of cancers. Knockdown of Mcl-1 with RNAi technology resulted in induced apoptosis and reduced growth of cancer cell lines. Campbell and coworkers determined that the overexpression of Mcl-1 in any cell line resulted in tumorigenesis. Increased regulation of this protein leads to a worse overall survival rate in the case of triple-negative breast cancers. Expression of mutant forms of BH3-mimicking proteins could bind to Mcl-1 and inhibit the metastasis and invasion of TN breast tumor cell lines in xenograft models (21). These results and findings suggest that these targets could be adapted for treatment options exclusively for breast cancer treatment.

Our results correlated with the results reported in the literature and it shows that our study is coherent with the previous work done with this protein. Mcl-1 was shown to have high levels of expression in untreated breast cancer cell lines. When treated with DF test compounds, the expression levels of Mcl-1 dropped significantly. These results show that plant compounds repress anti-apoptotic proteins’ expression, and complete silencing of Mcl-1 expression could be achieved with plant compounds. It is interesting to note that the compounds collectively down-regulate the anti-apoptotic proteins while up-regulating the pro-apoptotic proteins resulting in cell death. The physiological relevance and the mechanism of the regulation are yet to be ascertained in detail to know more about the impact of this protein with respect to the oncogenesis process.

***Bax and Bak-1***

Bcl-2-associated X protein (Bax) and Bcl-2 homologous antagonist/killer (Bak) are the primary members of the Bcl-2 family of pro-apoptotic proteins. Pro-apoptotic proteins can be classified into multidomain containing Bax and Bak and BH3-only proteins such as Bid, Bim, PUMA, Bad, Bik, and Hrk presence of Bcl-2 homology domains. Bax and Bak are primarily essential for forming mitochondrial outer membrane permeabilization (MOMP), helping in cytochrome C, a hallmark of apoptosis. Cells deficient in Bax and Bad were shown to not exhibit t-Bid mediated cytochrome-C release and apoptosis (27). Almost all anti-apoptotic proteins could inhibit Bax, whereas Bcl-xL, Mcl-1, and A1 primarily inhibit Bak. The effector BH3-proteins function either by direct binding to Bax and Bak, thus activating them, or indirectly by binding to anti-apoptotic proteins, thus freeing up Bax and Bak to form MOMP and triggering apoptosis (28).

***Bax***

Expression of Bax and Bax mutations plays a significant role in the resistance of cancer cells to apoptosis. Down-regulation of Bax in ovarian carcinoma cells resulted in enhanced cisplatin resistance. The Bax gene is found to be showing diminished regulation in various cancers including ovarian, breast, and pancreatic cancer. Bax expression is regulated by tumor suppressor p53 and is a vital part of the p53-mediated apoptosis. Reduced Bax levels are commonly observed in enhanced resistance towards chemotherapy in various cancers including chronic lymphocytic leukemia, prostate, liver, colorectal, and lung cancers (29, 30). Because of the ability to increase the apoptosis function and its low expression levels in cancer cell lines, both Bax and Bad are majorly focused on various kinds of cancer treatment strategies. Several anticancer agents which target apoptosis of cancer cells were designed to involve Bax. Apart from drugs that indirectly trigger Bax activation, direct activation of Bax by small molecules has started to gain traction in recent years. 

Our analysis found that Bax was present at low levels in untreated breast cancer cell lines. Upon treatment, DF-treated cell lines showed an increase with a two-fold increase in Bax expression. 

***Bak-1***

Bak-1 is characterized by four BH domains (BH1-4) and functions as a pro-apoptotic protein involved in several cellular processes. Bak-1 localizes to the mitochondrial outer membrane and stays in an inactive state unless stimulated by apoptotic signaling. When activated by effector BH3-proteins, Bak-1 stimulates mitochondrial pore formation, resulting in the cytochrome-c release, thus triggering apoptosis (28). Bak-1 overexpression leads to neurodegenerative and autoimmune disorders, whereas down-regulation leads to tumorigenesis. Bak-1 down-regulation has been reported in several cancers including, colorectal, gastric, and lung cancers. Targeted activation of Bak-1 and Bax has been investigated to stimulate apoptosis in several cancer cell lines (31). 

Our results revealed that the Bak-1 expression followed an almost similar profile compared to that of Bax. The massive change in expression levels of two similar proteins is particularly intriguing. DF showed a four-fold increase in expression. Up-regulation of Bak-1 and Bax of our test compounds resulted in enhanced apoptosis and cytotoxicity of cancer cells, thereby reiterating the chemical compounds’ potential to be used for cancer therapy.

***Bik***

The Bcl-2-interacting killer is a member of BH3-only proteins in the Bcl-2 family of apoptotic regulators. Bik is localized to the ER’s cytosolic membrane and is known to trigger apoptosis by transporting Ca^2+^ from ER to mitochondria and activation of Bax. Bik was known to interact with several anti-apoptotic proteins such as Bcl-2, Bcl2-L1, and E1B-19K and strongly trigger apoptosis when overexpressed (32). Several reports show the tumor suppressor activities of Bik. Bik was up-regulated in normal kidney epithelial cells, whereas in renal cell carcinomas, the Bik gene was knocked down by epigenetic promoter silencing (33). 

Bik was overexpressed in several cancerous cell lines such as lung, prostate, and renal carcinoma when treated with DNA methyltransferase 1 inhibitor and histone deacetylase inhibitor. An increase in Bik expression was correlated with enhanced apoptosis rates in cancer cell lines. Several mechanisms such as genome deletions, silencing of Bik gene expression, and post-transcriptional knockdown was done by regulating the Bik gene (34). Contrastingly, high levels of Bik were observed in sporadic breast tumors. In a study by Lu *et al.,* high Bik levels were associated with worsened prognosis of non-small cell lung cancers (35). Bik’s overexpression was correlated with up-regulation of caspase-8 and caspase-10, thus affirming that several mechanisms regulated the pro-apoptotic and anti-apoptotic roles of Bik (34). 

Our results revealed both up-regulation and down-regulation of Bik protein upon treatment with DF. DF showed a 4-fold increase in the expression. Since Bik can either act as a pro-apoptotic or anti-apoptotic regulator and was previously shown to be highly expressed in breast cancer cell lines, further studies are required to pinpoint the effect of the differential regulation of Bik when treated with test compounds. 

***Bid and bad***

BH3-interacting-domain death agonist (Bid) and Bcl-2 associated death promoter (Bad) are members of the pro-apoptotic Bcl-2 protein family characterized by the presence of only BH3 domains. Bid’s primary function is to interact with Bax and recruit Bax to the outer mitochondrial membrane to form MOMP leading to cytochrome C’s release, resulting in apoptosis. Chen *et al.* revealed that Bid, Bim, and Puma are effective apoptotic inducers in fibroblasts. At the same time, Bad and Noxa are weaker when expressed separately but are more efficient in killing cells. Bid, Bim, and Puma were known to activate Bax-Bak to enable cytochrome c release (36). The activator BH3 proteins such as Bid, Bim, Bad, and Puma are sequestered by anti-apoptotic Bcl-2 proteins such as Bcl-2, Bcl-xL, and Mcl-1, thus preventing the formation of MOMP and disabling cytochrome c release. Bad binds to Bcl-2 and Bcl-xL proteins, thus releasing Bid, Bim, and Puma, which might further lead to Bax-Bak activation. Protein kinases usually phosphorylate Bad. During stress conditions, Bad is dephosphorylated and moves to mitochondria, where it activates Bax-Bak, thus triggering apoptosis (36, 37). 

Bid and Bad, being pro-apoptotic proteins, were shown to be continuously down-regulated in several cancer cell lines. Bid and Bad were involved in enhancing the effectiveness of chemotherapeutic agents on cancer cells in mice (38). BH3-mimetic agents have been widely used as anticancer drugs. ABT-737 can bind to Bcl-2 anti-apoptotic proteins like Bad but cannot activate Bax and Bak (39). Bid expression was associated with enhanced tumor cell apoptosis in colon cancer cell lines, while Bad expression had no significant effect. The frequent phosphorylation of Bad could be the reason for its negligible effect on apoptosis. Ranger *et al.* observed spontaneous tumorigenesis in Bad deficient mice, which died due to radiation-induced thymic lymphomas much quicker than wild-type mice. Bid-deficient mice generated a clonal malignancy like human chronic myelomonocytic leukemia (40). Thus, reduced expression of Bid and Bad is generally correlated with poor prognosis in most cancers. 

Based on the results obtained from the 2D-gel analysis, Bid was found to be unanimously up-regulated when treated with all the test compounds. DF showed a 9-fold increase in expression. Higher Bid levels in breast cancer cell lines could be correlated with increased apoptotic rates in these cells. Similar to Bid, Bad was observed to be massively up-regulated with all compounds tested. 

***Beclin-1 N1***

Beclin-1, also called autophagy-related protein (ATG6), is a BH-3 domain-only protein and part of the Bcl-2 protein family. Beclin 1 interacts with Bcl-2 and neutralizes the anti-apoptotic activity of Bcl-2 (41). Beclin 1 functions as a potent autophagy-regulating protein. The role of autophagy, as well as Beclin-1 in tumorigenesis and malignant transformation, is heavily debated. Autophagy could act on cells with genetic defects, thus deterring them from malignant transformation and acting as a tumor suppressor. However, cancer cells get energy from angiogenesis and aerobic glycolysis during nutrient starvation and hypoxia conditions. 

Beclin-1 is characterized by the presence of the BH3 motif, coiled-coil domain, along with an evolutionarily conserved domain. Beclin-1, being a BH3-only protein, can self-oligomerize as well as interact with several Bcl-2 proteins. Beclin-1 at the protein level is regulated by cleaving proteins such as Calpain, caspase-3, caspase-7, and caspase-8. Cleavage of Beclin-1 by caspase-8 resulted in the formation of Beclin-1 1-N and Beclin-1 1-C truncated proteins that result in reduced autophagy. The truncated fragments do not exhibit autophagy but are known to promote pro-apoptotic functions (42). 

Beclin-1 was widely reported to have tumor-suppressive roles by several mechanisms, including autophagy of oncogenic proteins, activation of oncogene-induced senescence, anti-inflammatory responses, genetic stability, cell maintenance, and anti-microbial effects. Beclin-1 deficient mice developed lymphoma, lung carcinoma, and liver carcinoma (43). A single allele loss of Beclin-1 was observed in 40–75% of breast, prostate, and ovarian tumors, and a severely reduced expression of Beclin-1 was observed in prostate, ovarian, colon, brain, breast, and liver cancers (44). Knockdown of Beclin-1 by siRNA resulted in blocked spontaneous senescence in human fibroblasts. Reduced levels of Beclin-1 were associated with increased amounts of mutant p53, and when overexpressed, Beclin-1 reduced the amounts of mutant p53 (45). 

The up-regulation of the cleaved Beclin-1 N1 can be correlated with the results obtained for caspase-8 and to the up-regulation of caspase-8. Cleavage of Beclin-1 to Beclin-1 N1 should result in reduced autophagy. Autophagy suppression usually results in enhanced chemotherapy-induced apoptosis in the treated cell lines. Hence, the up-regulation of the cleaved BECN1 could affect high levels of caspase-8, which could direct the cells toward chemotherapy-induced apoptosis rather than autophagy. 

***Caspase 8***

Caspase 8 is a member of cysteine-aspartic acid proteases that have essential roles in apoptosis. Caspases are proteolytic enzymes that explicitly cleave the target protein after an aspartic acid residue and always exist in the cell in the inactivated form (pro-caspases). Caspases play significant roles in apoptosis, pyroptosis, necroptosis, cell proliferation, cell differentiation, and tumor suppression. Reduced levels of caspases were generally observed in several cancers. Caspase 8 was known to interact with the Bcl-2 family of proteins, including Bcl-2, Beclin-1, and Bid (46). 

Expression of caspases was shown to vary across different cancers. Caspase-3 overexpression was seen in acute myelogenous leukemia compared to normal cells (47). Contrastingly, poorly differentiated prostate cancer tissues showed low levels of caspase-3 when compared to well-differentiated cancer tissues. Knockdown of caspase-8 at mRNA and protein levels was observed in small-cell lung cancers, pediatric neuroblastoma, and neuroendocrine lung cancers (48). Teitz *et al.* revealed that caspase-8 could be a potent tumor suppressor in lung cancer and neuroblastoma (49). In breast cancer cell lines, chemotherapeutic resistance was correlated with dysregulation of caspase activity. While high caspase-3 expression was associated with adverse survival rates, no correlation was found between caspase-8 levels and breast cancer-specific survival (50). As discussed earlier, caspase-8 was also reported to cleave Beclin-1, thus suppressing autophagy, and directing the cells towards enhanced chemotherapy-induced apoptosis (51).

Like other pro-apoptotic proteins, caspase-8 was up-regulated severely in the treated cell lines. DF exhibited around a 10-fold increase in expression. Up-regulated caspase-8 might result in Bid’s cleavage to produce tBid, a vital regulator essential for MOMP formation and cytochrome C release. Caspase-8 could also cleave Beclin-1 and account for the increased levels of Beclin-1 N-1 fragment in the test samples. 

Altogether, based on our results, the DF compound greatly up-regulates pro-apoptotic proteins’ expression while down-regulating pro-survival/anti-apoptotic proteins in breast cancer cell lines, thus showing strong promise to be considered an anticancer agent for breast cancer chemotherapy.

## Conclusion

The current study observed that the *D. falcata *plant extract has the potential to induce apoptotic proteins in breast cancer cells. Proteome-wide analysis found that apoptotic pathway proteins including BAD, BIK, BID, CASP8, MCL1, BCL2, and BAK1 were highly impacted by treatment with the *D. falcata *plant extract. All these protein hits were validated by RT PCR analysis which further confirms their significant impact in altering the apoptotic pathway in breast cancer cells. All of these validated key proteins possess important roles in inducing the apoptosis process which is an essential necessity in the cancer treatment target process. Initiation of these apoptotic proteins by *D. falcata *plant extract treatment in breast cancer cells shows us a positive direction towards nature-based alternative medicine.

## Authors’ Contributions

All authors equally contribute to this research article.

## Data Availability

The data used to support the findings of this study are included in the article.

## Ethical Approval

This article does not contain any studies with human participants or animals performed by any of the authors.

## Conflicts of Interest

The authors declare that they have no conflicts of interest. 

## References

[1] Agarwal G, Pradeep PV, Aggarwal V, Yip CH, Cheung PS (2007). Spectrum of breast cancer in Asian women. World J Surg.

[2] Altenberg B, Greulich KO (2004). Genes of glycolysis are ubiquitously overexpressed in 24 cancer classes. Genomics.

[3] Candiano G, Bruschi M, Musante L, Santucci L, Ghiggeri GM, Carnemolla B (2004). Blue silver: A very sensitive colloidal Coomassie G-250 staining for proteome analysis. Electrophoresis.

[4] Bray F, Ferlay J, Soerjomataram I, Siegel RL, Torre LA, Jemal A (2018). Global cancer statistics 2018: GLOBOCAN estimates of incidence and mortality worldwide for 36 cancers in 185 countries. CA Cancer J Clin.

[5] Capello M, Ferri-Borgogno S, Riganti C, Chattaragada MS, Principe M, Roux C (2016). Targeting the Warburg effect in cancer cells through ENO1 knockdown rescues oxidative phosphorylation and induces growth arrest. Oncotarget.

[6] Pattanayak SP, Mazumder PM, Sunita P (2011). Total phenolic content, flavonoid content and in vitro anti-oxidant activities of Dendrophthoe falcata (L f ) Ettingsh. Res J Med Plan.

[7] Nigam YP (2022). The bornean mistletoes as versatile parasites: A systematic review. Sys Rev Pharm.

[8] Mosmann T (1983). Rapid colorimetric assay for cellular growth and survival: application to proliferation and cytotoxicity assays. J Immunol Methods.

[9] Christensen MV, Hogdall CK, Jochumsen KM, Hogdall EVS (2018). Annexin A2 and cancer: A systematic review. Int J Oncol.

[10] Mi H, Thomas P, Nikolsky Y (2009). Panther pathway: An Ontology-Based Pathway Database Coupled with Data Analysis Tools. Protein Networks and Pathway Analysis. Methods in Molecular Biology.

[11] Szklarczyk D, Franceschini A, Wyder S, Forslund K, Heller D, Huerta-Cepas J (2015). STRING v10: protein-protein interaction networks, integrated over the tree of life. Nucleic Acids Research.

[12] Tsujimoto Y, Cossman J, Jaffe E, Croce CM (1985). Involvement of the bcl-2 gene in human follicular lymphoma. Science.

[13] Honma N, Horii R, Ito Y, Saji S, Younes M, Iwase T (2015). Differences in clinical importance of Bcl-2 in breast cancer according to hormone receptors status or adjuvant endocrine therapy. BMC Cancer.

[14] Teixeira C, Reed JC, Pratt MA (1995). Estrogen promotes chemotherapeutic drug resistance by a mechanism involving Bcl-2 proto-oncogene expression in human breast cancer cells. Cancer Res.

[15] Campbell KJ, Tait SWG (2018). Targeting BCL-2 regulated apoptosis in cancer. Open Biol.

[16] Loo LSW, Soetedjo AAP, Lau HH, Ng NHJ, Ghosh S, Nguyen L (2020). BCL-xL/BCL2L1 is a critical anti-apoptotic protein that promotes the survival of differentiating pancreatic cells from human pluripotent stem cells. Cell Death Dis.

[17] Lizarraga-Verdugo E, Ruiz-Garcia E, Lopez-Camarillo C, Bermudez M, Avendano-Felix M, Ramos-Payan R (2020). Cell survival is regulated via SOX9/BCL2L1 axis in HCT-116 colorectal cancer cell line. J Oncol.

[18] Ghaemi S, Arefian E, Rezazadeh Valojerdi R, Soleimani M, Moradimotlagh A, Jamshidi Adegani F (2020). Inhibiting the expression of anti-apoptotic genes BCL2L1 and MCL1, and apoptosis induction in glioblastoma cells by microRNA-342. Biomed Pharmacother.

[19] Sillars-Hardebol AH, Carvalho B, Belien JA, de Wit M, Delis-van Diemen PM, Tijssen M (2012). BCL2L1 has a functional role in colorectal cancer and its protein expression is associated with chromosome 20q gain. J Pathol.

[20] Park H, Cho SY, Kim H, Na D, Han JY, Chae J (2015). Genomic alterations in BCL2L1 and DLC1 contribute to drug sensitivity in gastric cancer. Proc Natl Acad Sci U S A.

[21] Campbell KJ, Dhayade S, Ferrari N, Sims AH, Johnson E, Mason SM (2018). MCL-1 is a prognostic indicator and drug target in breast cancer. Cell Death Dis.

[22] Akgul C, Turner PC, White MR, Edwards SW (2000). Functional analysis of the human MCL-1 gene. Cell Mol Life Sci.

[23] Wang JM, Lai MZ, Yang-Yen HF (2003). Interleukin-3 stimulation of mcl-1 gene transcription involves activation of the PU 1 transcription factor through a p38 mitogen-activated protein kinase-dependent pathway. Mol Cell Biol.

[24] Gong J, Zhang JP, Li B, Zeng C, You K, Chen MX (2013). MicroRNA-125b promotes apoptosis by regulating the expression of Mcl-1, Bcl-w and IL-6R. Oncogene.

[25] Inuzuka H, Shaik S, Onoyama I, Gao D, Tseng A, Maser RS (2011). SCF(FBW7) regulates cellular apoptosis by targeting MCL1 for ubiquitylation and destruction. Nature.

[26] Zhang S, Zhang M, Jing Y, Yin X, Ma P, Zhang Z (2018). Deubiquitinase USP13 dictates MCL1 stability and sensitivity to BH3 mimetic inhibitors. Nat Commun.

[27] Kazhdan I, Long L, Montellano R, Cavazos DA, Marciniak RA (2006). Targeted gene therapy for breast cancer with truncated Bid. Cancer Gene Ther.

[28] Kang MH, Reynolds CP (2009). Bcl-2 inhibitors: targeting mitochondrial apoptotic pathways in cancer therapy. Clin Cancer Res.

[29] Naseri MH, Mahdavi M, Davoodi J, Tackallou SH, Goudarzvand M, Neishabouri SH (2015). Up regulation of Bax and down regulation of Bcl2 during 3-NC mediated apoptosis in human cancer cells. Cancer Cell Int.

[30] Liu Z, Ding Y, Ye N, Wild C, Chen H, Zhou J (2016). Direct activation of bax protein for cancer therapy. Med Res Rev.

[31] Duckworth CA, Pritchard DM (2009). Suppression of apoptosis, crypt hyperplasia, and altered differentiation in the colonic epithelia of bak-null mice. Gastroenterology.

[32] Pandya V, Glubrecht D, Vos L, Hanson J, Damaraju S, Mackey J (2016). The pro-apoptotic paradox: The BH3-only protein Bcl-2 interacting killer (Bik) is prognostic for unfavorable outcomes in breast cancer. Oncotarget.

[33] Sturm I, Stephan C, Gillissen B, Siebert R, Janz M, Radetzki S (2006). Loss of the tissue-specific proapoptotic BH3-only protein Nbk/Bik is a unifying feature of renal cell carcinoma. Cell Death Differ.

[34] Chinnadurai G, Vijayalingam S, Rashmi R (2008). BIK, the founding member of the BH3-only family proteins: mechanisms of cell death and role in cancer and pathogenic processes. Oncogene.

[35] Lu Y, Lemon W, Liu PY, Yi Y, Morrison C, Yang P (2006). A gene expression signature predicts survival of patients with stage I non-small cell lung cancer. PLoS Med.

[36] Chen L, Willis SN, Wei A, Smith BJ, Fletcher JI, Hinds MG (2005). Differential targeting of prosurvival Bcl-2 proteins by their BH3-only ligands allows complementary apoptotic function. Mol Cell.

[37] Billen LP, Shamas-Din A, Andrews DW (2008). Bid: A Bax-like BH3 protein. Oncogene.

[38] Sinicrope FA, Rego RL, Foster NR, Thibodeau SN, Alberts SR, Windschitl HE (2008). Proapoptotic Bad and Bid protein expression predict survival in stages II and III colon cancers. Clin Cancer Res.

[39] Walensky LD, Kung AL, Escher I, Malia TJ, Barbuto S, Wright RD (2004). Activation of apoptosis in vivo by a hydrocarbon-stapled BH3 helix. Science.

[40] Ranger AM, Zha J, Harada H, Datta SR, Danial NN, Gilmore AP (2003). Bad-deficient mice develop diffuse large B cell lymphoma. Proc Natl Acad Sci U S A.

[41] Vega-Rubin-de-Celis S (2019). The role of beclin 1-dependent autophagy in cancer. Biology (Basel).

[42] Li H, Wang P, Sun Q, Ding WX, Yin XM, Sobol RW (2011). Following cytochrome c release, autophagy is inhibited during chemotherapy-induced apoptosis by caspase 8-mediated cleavage of Beclin 1. Cancer Res.

[43] Aita VM, Liang XH, Murty VV, Pincus DL, Yu W, Cayanis E (1999). Cloning and genomic organization of beclin 1, a candidate tumor suppressor gene on chromosome 17q21. Genomics.

[44] Yue Z, Jin S, Yang C, Levine AJ, Heintz N (2003). Beclin 1, an autophagy gene essential for early embryonic development, is a haploinsufficient tumor suppressor. Proc Natl Acad Sci U S A.

[45] Jung YY, Lee YK, Koo JS (2016). The potential of Beclin 1 as a therapeutic target for the treatment of breast cancer. Expert Opin Ther Targets.

[46] Fritsch M, Gunther SD, Schwarzer R, Albert MC, Schorn F, Werthenbach JP (2019). Caspase-8 is the molecular switch for apoptosis, necroptosis and pyroptosis. Nature.

[47] Estrov Z, Thall PF, Talpaz M, Estey EH, Kantarjian HM, Andreeff M (1998). Caspase 2 and caspase 3 protein levels as predictors of survival in acute myelogenous leukemia. Blood.

[48] Winter RN, Kramer A, Borkowski A, Kyprianou N (2001). Loss of caspase-1 and caspase-3 protein expression in human prostate cancer. Cancer Res.

[49] Teitz T, Wei T, Valentine MB, Vanin EF, Grenet J, Valentine VA (2000). Caspase 8 is deleted or silenced preferentially in childhood neuroblastomas with amplification of MYCN. Nat Med.

[50] Pu X, Storr SJ, Zhang Y, Rakha EA, Green AR, Ellis IO (2017). Caspase-3 and caspase-8 expression in breast cancer: Caspase-3 is associated with survival. Apoptosis.

[51] Wirawan E, Vande Walle L, Kersse K, Cornelis S, Claerhout S, Vanoverberghe I (2010). Caspase-mediated cleavage of Beclin-1 inactivates Beclin-1-induced autophagy and enhances apoptosis by promoting the release of proapoptotic factors from mitochondria. Cell Death Dis.

